# Compound heterozygous *FAM20C* gene variants in a patient with severe Raine syndrome: a case report

**DOI:** 10.3389/fgene.2023.1179163

**Published:** 2023-04-26

**Authors:** Camelia Chirteș, Alina Bogliș, Andrea Toth, Corina Rac, Claudia Bănescu

**Affiliations:** ^1^ Laboratory of Genetics, Department of Genetics, Emergency County Hospital, Târgu Mureș, Romania; ^2^ Department of Genetics, George Emil Palade University of Medicine, Pharmacy, Science and Technology, Târgu Mureș, Romania; ^3^ Department of Neonatology, Emergency County Hospital, Târgu Mureș, Romania; ^4^Center for Advanced Medical and Pharmaceutical Research, George Emil Palade University of Medicine, Pharmacy, Science and Technology of Targu Mures, Târgu Mureș, Romania

**Keywords:** case report, raine syndrome, craniofacial dysmorphism, osteosclerosis, *FAM20C* gene, congenital disorder

## Abstract

Raine syndrome is a congenital disorder caused by biallelic mutations in the *FAM20C* gene. While most diagnosed cases of the syndrome are lethal in the first few months of life, there are also reports of non-lethal cases with Raine syndrome. The characteristic of this syndrome is typical facial dysmorphism and generalized osteosclerosis, as well as possible intracranial calcification, hearing loss, and seizures. We report a case of a 4-day-old patient at the time of examination, born with a distinct facial dysmorphism, short neck, narrow chest, and curved tibia. The parents, affirmative gypsy and non-consanguineous, had a previous male child born with the same phenotype who died at 4 months old. The computed tomography scan revealed choanal atresia, while transfontanelar ultrasound showed hypoplasia of the frontal and temporal lobes, corpus callosum dysgenesis, and multiple areas of intracranial hyperechogenicity. The chest X-Ray revealed generalized increased bone density. A skeletal disorders gene panel was performed which identified two variants in the *FAM20C* gene: a pathogenic variant c.1291C>T (p.Gln431*) and a likely pathogenic variant (c.1135G>A) (p.Gly379Arg), confirming the clinical diagnosis. The parents were also tested, and each was found to carry one of the variants. The particularity of this case is the severe phenotype in a compound heterozygous case that consists of *FAM20C* c.1291C>T (p.Gln431*) variant that has recently been reported in the literature. Also, our case is one of the few compound-heterozygous mutations in the *FAM20C* gene that has been described in a non-consanguineous marriage.

## 1 Introduction

Raine syndrome (MIM #259775) is an autosomal recessive disorder caused by a mutation in the *FAM20C* gene. To the present day, two phenotypes of the syndrome have been reported: lethal and non-lethal Raine syndrome. Lethal Raine syndrome is highly aggressive with a low survival rate, while non-lethal Raine syndrome has a much better survival rate, a milder form of symptomatology, with the oldest diagnosed patient being 72 years old (Bajaj, S., et al., 2021). While there is no clear definition of non-lethal Raine syndrome, the described cases survived during infancy and were living a normal life, with milder or no clinical dimorphism but all of them presented bone disorders. Simpson et al. (2009) were the first to report on non-lethal Raine syndrome when the patients were 9 and 11 years old. Approximately 70 cases of Raine syndrome (40 cases of lethal Raine Syndrome and 30 non-lethal cases) have been reported (Xu, R., et al., 2021), with a prevalence of 1/100,000 cases, requiring continuous investigations to understand better the syndrome, its functioning mechanism, and possible treatment (Rameh, G., et al., 2022). Raine et al. described in 1989 an unknown multiple congenital anomalies syndrome in a newborn who died soon after birth, later on, identified as Raine Syndrome (Hernández-Zavala, A., et al., 2020). The following described cases also had a very short survival with an early and aggressive onset, making the syndrome considered lethal (Al Mane et al., 1998). In the following years, non-lethal Raine syndrome cases also appeared with the increase in number of reported cases. The syndrome’s characteristic phenotype includes craniofacial dysmorphism such as microcephaly, brachycephaly, wide open anterior and posterior fontanels, intracranial calcification, mid-face hypoplasia, proptosis, micrognathia, high and/or cleft palate, hypoplastic nose, low set ears (Hung, C.Y., et al., 2019) generalized osteosclerosis with periosteal formation, fracture-like ribs. (Elalaoui, S.C., et al., 2016).

It is known that Raine syndrome is caused by a mutation in the family with sequence similarity 20, member C (*FAM20C*) gene. The *FAM20C* gene, which is located on chromosome 7p22.3, encodes a protein that binds calcium and phosphorylates proteins involved in bone mineralization. Recent studies suggested that the *FAM20C* gene also suppresses fibroblast growth factor (FGF 23) production by enhancing dentin matrix protein 1 (DMP1) expression, inactivating mutation in the gene causing FGF23-related hypophosphatemia (Eltan, M., et al., 2020). Our aim is to present a compound heterozygous newborn with a severe phenotype diagnosed with Raine syndrome from non-consanguineous parents.

## 2 Materials and methods

### 2.1 Ethical compliance

The parents provided written informed consent for genetic analysis from peripheral blood and data collection. Additionally, written informed consent was obtained from the child’s legal guardian to publish photos in this article. The study was approved by the Ethics Committee of Emergency County Hospital, Târgu Mureș (nr.ad.3310 from 10.02.2023).

### 2.2 Case description

We report a case of a 4-day-old male patient born to a healthy non-consanguineous couple, with the mother being 19 years old G2P2A0 (Gravidity2, Para2, Abortions0) and the father being 21 years old. There was no reported history of similar cases in the family. However, the couple had lost a male child with similar clinical features at 4 months old, a year before. The Family pedigree can be found in the Supplementary Materials. Although the first-trimester screening was normal, an additional investigation was recommended due to the family history. The non-invasive prenatal test (NIPT) was performed with normal results, but follow-up fetal/prenatal ultrasound (US) examinations revealed multiple malformations requiring further investigations. Despite this, the mother did not seek medical care until a few weeks before the onset of labor. At 37 weeks of gestation, the fetal ultrasound scan showed polyhydramnios and signs of fetal distress. Thus, a C-section was performed, and at delivery, the patient exhibited abnormal breath sounds, marked cyanosis, multiple congenital anomalies, and a birth weight of 3500 g, a length of 52 cm, and head circumference of 35 cm, Apgar score of 5 and 5 at 1- and 5-min. Mechanical ventilation was unsuccessful at birth due to anatomical abnormalities. Considering the severe respiratory distress with desaturations up to 60% caused by the choanal atresia and pulmonary hypoplasia, mechanical ventilation succeeded. During the first month of the patient’s life, a tracheostomy was performed. A few days after birth, the patient developed opisthotonos, a rigid posture that has remained constant. Despite severe delays in psychomotor development, the patient has been able to gain weight and height.

**FIGURE 1:**
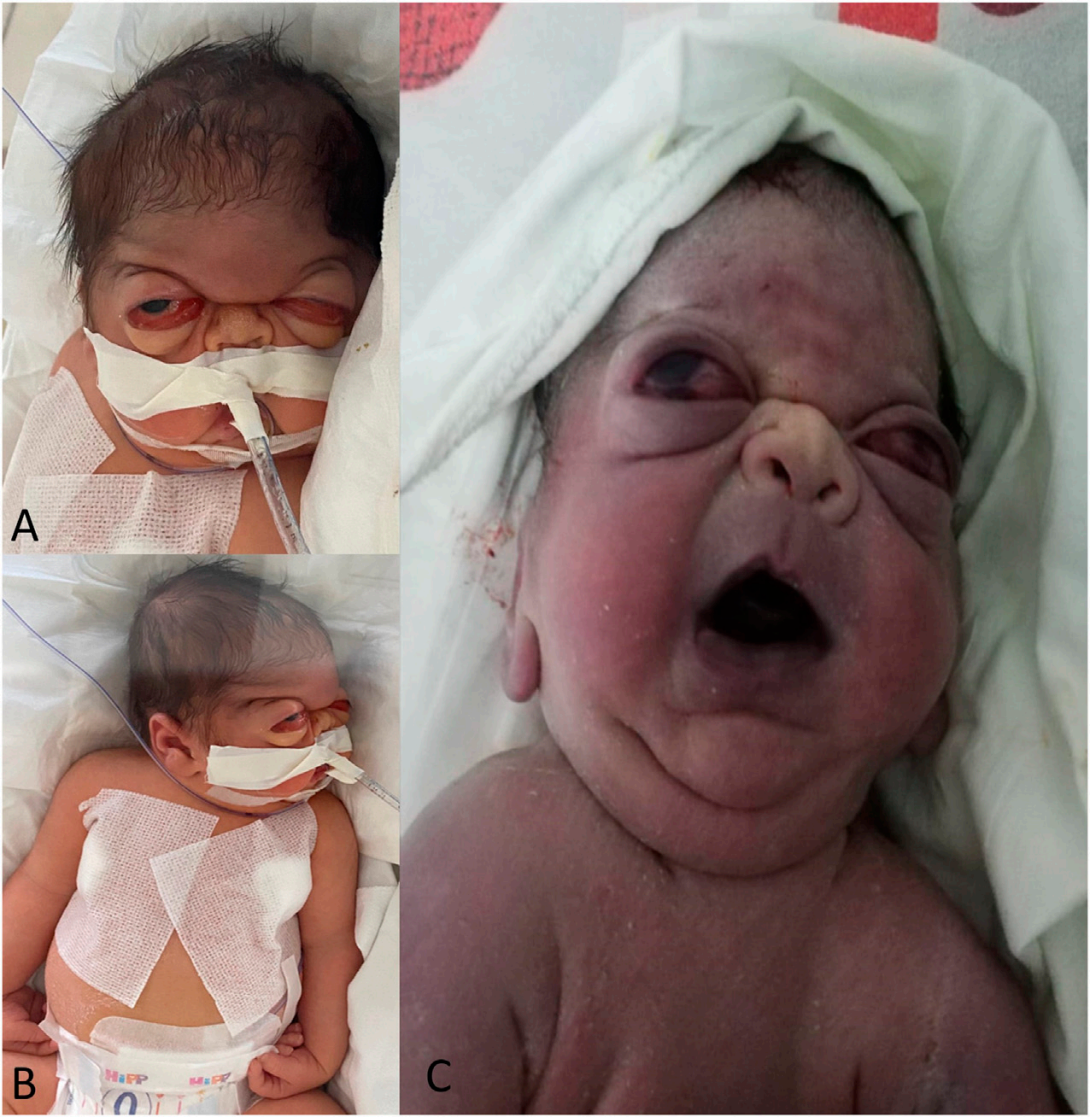
The patient displays distinct craniofacial dysmorphism, including severe bilateral proptosis, frontal bossing, low-set ears, and a depressed nasal bridge **(A)**. The patient has an abnormal wrist position with bilateral wrist flexion contracture, and bilateral finger contracture **(B)**. The proband’s brother presented similar clinical features in the first days after birth, including severe proptosis, depressed nasal bridge, triangularly shaped mouth, short neck, and low-set ears **(C)**.

The couple’s first-born child was also a male delivered by C-section at 37 weeks of gestation, with a birth weight of 2800 g. The infant exhibited several clinical features, including cyanosis, dysmorphic facial features, wide-open fontanelles, and an anterior fontanelle measuring 3/3.5 cm. Additionally, the infant presented a craniofacial dysmorphism with trigonocephaly, midface hypoplasia, choanal atresia, ectropion, severe proptosis, a depressed nasal bridge, a triangularly-shaped mouth, low-set ears, a short neck (Figure 1C), a narrow chest, and severe respiratory distress, necessitating immediate mechanical ventilation after birth.

### 2.3 Imagistic investigation

**FIGURE 2:**
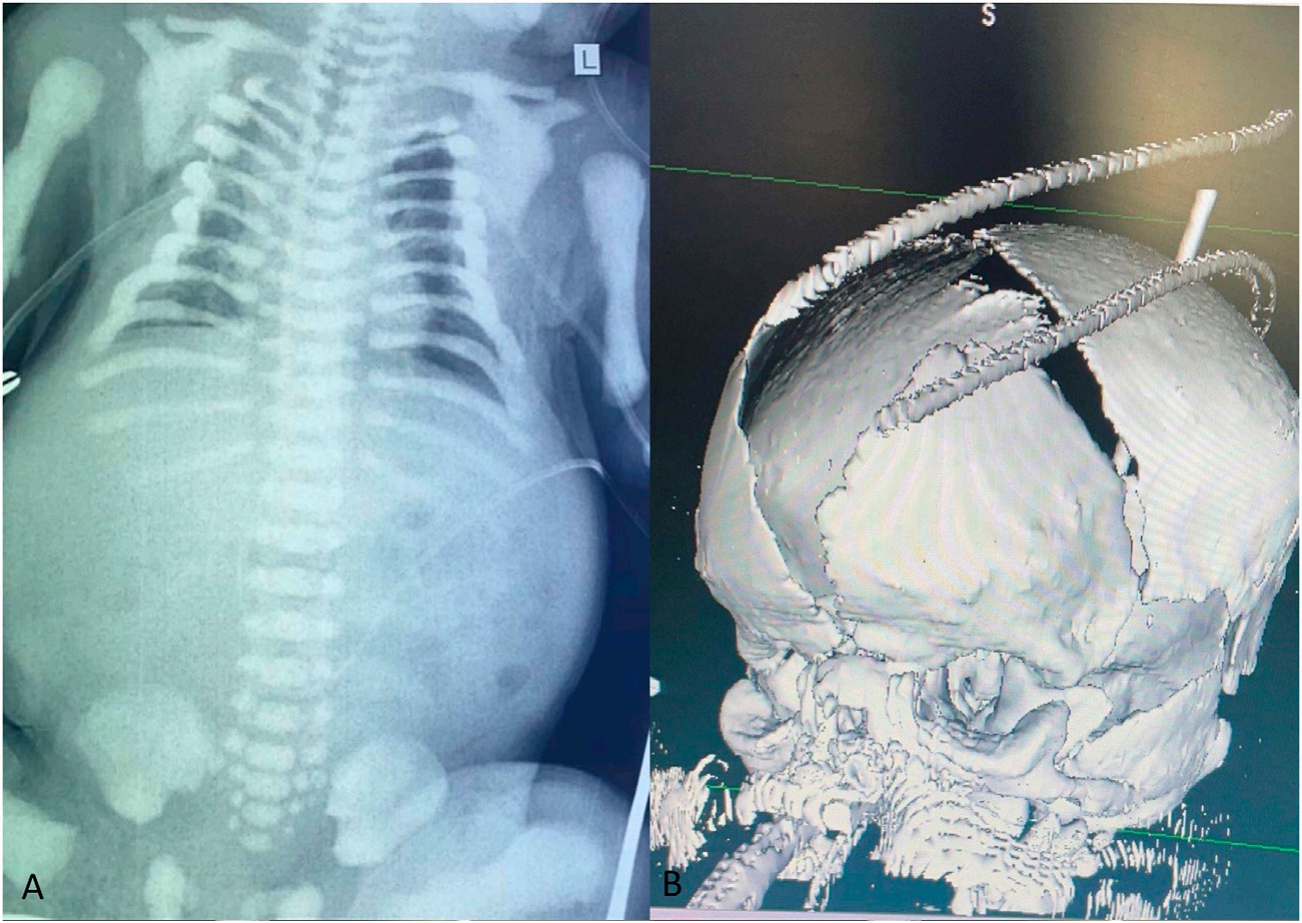
The chest X-ray in anteroposterior view indicates increased bone density in the ribs, vertebrae, scapulae, clavicles, humeri, and pelvis, which suggests the presence of generalized osteosclerosis **(A)**. The brain three-dimensional CT scan reveals a widely open anterior fontanelle **(B)**.

The patient’s chest X-ray revealed the presence of multiple bone calcifications, while abdominal ultrasonography showed mild bilateral pyelectasis (Figure 2A). Cranial computed tomography (CT) scan showed periventricular calcifications, cerebral atrophy, and hypoplasia of the corpus callosum. A three-dimensional brain CT scan was also conducted, which displayed an enlarged anterior fontanelle and larger than expected soft spots for the baby’s age (Figure 2B). Furthermore, echocardiography revealed contractility in the border zone. Ophthalmological examination revealed hyperemic conjunctivitis, bilateral palpebral edema, and mild corneal edema. Based on these imaging findings, which suggested generalized osteosclerosis and craniofacial dysmorphism, a possible diagnosis of Raine Syndrome was considered. Subsequently, we conducted further investigations with the biochemical evaluation. These investigations revealed an elevated serum parathyroid hormone (PTH) level (54.72 pg/mL; reference range: 5.7–34), low vitamin D level (16.10 ng/mL; reference range: 30–65), and normal serum calcium level (2.14 mmol/L; reference range: 1.90–2.60).

### 2.4 Genetic testing

Considering the severe phenotype cytogenetic analysis from blood was performed with a normal result (46,XY). Furthermore, due to severe craniofacial dysmorphia, the multiplex ligation-dependent probe amplification (MLPA) analysis was performed in our laboratory using the SALSA MLPA probemix P080 craniofacial assay (MRC Holland, Amsterdam, Netherlands). However, MLPA analysis did not identify any deletions or duplications in genes such as *FGFR1, FGFR2, FGFR3, TWIST1, MSX2, ALX1, ALX3, ALX4, RUNX2*, and *EFNB1*. Moreover, MLPA analysis did not detect any point mutations in the *FGFR3* gene (c.749C>G) or the presence of the c.755C>G wild-type sequence in the *FGFR2* gene. Next-generation sequencing using a Skeletal Disorders Panel was conducted to analyze the proband’s molecular profile, examining/testing up to 358 genes, and targeted sequencing was performed for the parents (Invitae, San Francisco, CA, United States). Genomic DNA isolated from a blood sample of the patient was enriched for targeted regions by applying a protocol based on hybridization. For sequencing, Illumina technology was used. The raw reads were aligned to a reference sequence, namely, Genome Reference Consortium Human Build 37 (GRCh37) also known as human genome assembly 19 (hg19).

## 3 Results

Upon clinical examination 4 days after birth, the newborn displayed craniofacial dysmorphism. The observed characteristics included a wide-open anterior fontanelle measuring 5/6 cm, trigonocephaly, frontal bossing, severe proptosis, a depressed nasal bridge, a small nose with narrow nares, bilateral choanal atresia, a large earlobe, a prominent antitragus, a small and triangularly-shaped mouth, microretrognathia, a superior mouth cleft, an inferior lip pseudo cleft, low-set ears, a short neck, a narrow thorax, and bilateral bowed tibia (Figures 1A, B).

Our patient has been identified as a compound heterozygote for two variants in the *FAM20C* gene, namely, the pathogenic heterozygous variant c.1291C>T (p.Gln431*) and the likely pathogenic heterozygous variant (c.1135G>A) (p.Gly379Arg). Testing of the parents revealed that each of them carried one of these variants. The mother carried the pathogenic variant (c.1135G>A) (p.Gly379Arg), while the father carried the other variant c.1291C>T (p.Gln431*).

The variant in *FAM20C* (c.1135G>A) (p.Gly379Arg) gene, also referred to as 1093G>A (Gly365Arg) or rs267606795, is not found in population databases such as Genome Aggregation Database (gnomAD). SIFT algorithm predicts that this variant is “damaging” with a score of 0.001. Based on the guidelines provided by the American College of Medical Genetics (American College of Medical Genetics, 2022) and VarSome, (Kopanos et al., 2019), this variant is classified as pathogenic. Additionally, Mutation Taster and MutPred2 software (Pejaver et al., 2020) both predict that this variant is disease-causing and pathogenic, respectively.

Moreover, we used the MutPred2 software, a machine learning-based method that integrates genetic and molecular data to quantify the pathogenicity of amino acid substitution in our case. For the *FAM20C* c.1135G>A (p.Gly379Arg; G379R) substitution, MutPred2 predicted changes in metal binding and transmembrane protein, as well as a loss of disulfide linkage at C378, with a MutPred2 score of 0.916 and *p*-values ≤0.05.

The *FAM20C* c.1291C>T (p.Gln431*) variant, which causes a premature translational stop signal leading to disrupted protein production, has recently been added to human variant databases such as ClinVar (National Center for Biotechnology Information, 2022. ClinVar) and is classified as pathogenic by VarSome (Kopanos et al., 2019) and likely pathogenic according to ACMG. However, it was not found in databases such as Genome Aggregation Database, 2022 (gnomAD), 1000 Genomes Project, 2022 (1000G), or Ensembl, 2022. The Mutation Taster predicts that the *FAM20C* c.1291C>T (p.Gln431*) variant is disease-causing. In addition, we used MutPred-LOF (Kymberleigh et al., 2017), a machine learning-based method and software, to predict the effect of the identified stop gain variant in our patient. For the *FAM20C* c.1291C>T (p.Gln431*; Q431*) non-sense mutation, also referred to as a loss of function variant, MutPred-LOF predicts the following molecular alterations that interest iron binding (*p* = 0.0001); catalytic site (*p* = 0.00023); PPI hotspot (*p* = 0.0007); sulfation (*p* = 0.003); signal helix (*p* = 0.005).

Table 1 presents the location, origin, and molecular consequences of the *FAM20C* variants found in our patient.

**TABLE 1:** The compound heterozygous variant of the FAM20C gene in our patient.

Location (GRCh38)	*FAM20C* variant	Protein change	Exon	Mutation Taster/PolyPhen-2	Kinase domain localization	VEP (variant effect predictor) impact	Molecular consequence	Classification of ACMG	Origin of *FAM20C* variants
chr7: 256691	c.1291C>T p.Gln431* or p.Gln431Ter	Q431*	7	Disease causing, score 0.81/	+	High/modifier	Non-sense	Likely pathogenic	Paternal
chr7: 255911	c.1135G>A p.Gly379Arg	G379R	6	Disease causing, score 0.81./probably damaging score = 1.0	+	Moderate/modifier	Missense	Pathogenic	Maternal

PolyPhen-2 - is a predictive tool that is utilized to assess the potential functional impact of missense mutations on the structure and function of the protein produced by the FAM20C gene (http://genetics.bwh.harvard.edu/pph2/). VEP (variant effect predictor) http://www.ensembl.org/Homo_sapiens/Tools/VEP

The patient has been under follow-up care since birth. Due to choanal atresia and severe respiratory distress, the patient underwent a tracheostomy in the first month of life. Since then, the patient has been frequently hospitalized for respiratory infections and failure to thrive, with a *Klebsiella* infection at 7 months of age. Non-etheless, the patient’s growth has improved and stabilized. At 4 months of age, the patient was diagnosed with acute gastritis and cerebral vasculitis. The patient is currently fed through a nasogastric tube and still relies on a tracheostomy tube for breathing. Neurological examination indicates hyperextension of the head-opisthotonus, diminished muscle tone and reflexes, and developmental delay in neuro psychomotor skills. The patient, now nearly one-year-old, is under institutionalized pediatric hospital care, and there has been no significant change in his health status. The most recent auxological measurements show a weight of 6500 g and a height of 68 cm.

## 4 Discussion

The initial cases of Raine syndrome reported in the literature exhibited severe symptoms and had a very low survival rate, with death occurring before birth or during the neonatal period due to severe respiratory failure. These cases were later identified as the lethal Raine syndrome (Hülskamp, G., et al., 2003). However, several non-lethal cases of Raine syndrome have been documented in recent years, clarifying the distinction between lethal and non-lethal types.

This disorder is characterized by typical craniofacial and skeletal modifications, including microcephaly/trigonocephaly, exophthalmos, hypoplastic nose, cleft palate/uvula, low-set ears, severe midface hypoplasia with choanal atresia, and generalized osteosclerosis (Mameli, C., et al., 2020). Most reported cases of Raine syndrome involve patients born at or near term who exhibit multiple phenotypic modifications (Ababneh, F.K., et al., 2013). Whether the syndrome is lethal or non-lethal is a determining factor for patient survival. Infants with the lethal form typically die during their first year of life due to complications such as choanal atresia, retrognathism, narrow chest, and pulmonary hypoplasia (Ishikawa, H.O., et al., 2012). Those who survive the newborn period are classified as having the non-lethal form (Mamedova, E., et al., 2019), and the survival rate for patients with Raine syndrome has been increasing due to improved recognition of clinical signs and supportive therapy options (Simpson, M.A., et al., 2009). Reported cases have ranged in age from the first few days of life to 27 years of age, with only two elderly cases at ages 61 and 72 presenting a wide range of symptoms depending on the form of Raine syndrome (Takeyari, S., et al., 2014). Most cases of Raine syndrome that are not lethal are characterized by discrete craniofacial dysmorphism, including a flat forehead, hypertelorism, epicanthal folds, a depressed nasal bridge, and a prominent philtrum. In addition to these features, some non-lethal cases have been reported to have short stature (Hrist et al., 2021) and severe motor developmental delay (Sheth, J., et al., 2018). These patients also presented seizures at a young age and bone malformations such as short fingers, under-mineralized distal phalanges, bowing of the long bones, metaphyseal flaring, and thoracic hypoplasia. (Simpson, M.A., et al., 2017). *FAM20C* is a member of the “family with sequence similarity 20” along with *FAM20A* and *FAM20B*, contributing to the organism’s physiological process among O-mannosyl kinase and the vertebrate lonesome kinase (VLK). (Acevedo, A.C., et al., 2015). *FAM20C* gene encodes a Golgi casein kinase protein. *FAM20C*, also known as Dentin Matrix Protein 4 (DMP4) or G-CK Golgi Casein Kinase FAM20C, is highly expressed in mineralized tissues and has a crucial role in normal bone mineralization, being known as a kinase for fibroblast growth factor 23 (FGF23). Mutations in the *FAM20C* gene have been linked to growth retardation and errors in osteoblast differentiation (Simpson, M.A., et al., 2017). In Raine syndrome, all the observed phenotypic modifications are caused by mutations in the *FAM20C* gene. The variability of phenotype in Raine Syndrome may be produced by changes in the expression of *FAM20C* protein generated by distinctive pathogenic variants. Different pathogenic variants can produce variability in phenotype severity, affecting the folding and secretion of kinase activity or the complete lack of it. Besides its role in bone tissues, FAM20C is also essential in non-bone tissues, acting on neuropeptides of secretory pathways in nervous and endocrine systems, vascular calcifications through sortilin1, and metabolism through its targets PCSK7 and PCSK9. While some suggest its potential role in redox homeostasis, clotting, and salivary glands, no clinical evidence supports it (Palma-Lara, I., et al., 2021). According to available information, 53 distinct variations (identified as pathogenic by ClinVar) have been documented in cases of Raine syndrome, encompassing splicing defects, frameshift, missense, non-sense mutations (causing truncated proteins), and chromosomal rearrangements, both lethal and non-lethal. In addition to the variant found in our case c.1291C>T (p.Gln431*), the variant c.1135G>A (p.Gly379Arg) was identified in one lethal homozygous case and one non-lethal case, possibly associated with severe cases of Raine syndrome (Xu et al., 2021). While consanguinity was typically observed in most cases, our case involved parents who were not related through consanguineous marriage (Mameli, C.,2020).

Given that the couple’s first child passed away at the age of 4 months and that the second child described in this manuscript is particularly severe and requires ongoing hospital care, it is likely that the child has only survived up to the age of 1 year due to the severity of the condition. We may consider our case as lethal Raine syndrome.

Since the second child was also diagnosed with Raine syndrome caused by biallelic variations in the *FAM20C* gene, the family received genetic counseling related to compound heterozygosity and autosomal inheritance pattern to assess the risk of transmission in future pregnancies.

## 5 Conclusion

We report compound heterozygous variants of the FAM20C gene in a patient with Raine syndrome, with one of the variants being the c.1291C>T (p.Gln431*) that has recently been described in the literature from non-consanguineous parents. Any neonate or infant with craniofacial and skeletal abnormalities such as microcephaly/trigonocephaly, exophthalmos, hypoplastic nose, cleft palate/uvula, low-set ears, severe midface hypoplasia with choanal atresia, and generalized osteosclerosis should raise suspicion of Raine syndrome.

## Data Availability

The datasets for this article are not publicly available due to concerns regarding participant/patient anonymity. Requests to access the datasets should be directed to the corresponding author.

## Abbreviations

FAM20C, family with sequence similarity 20; FGF23, growth factor 23; DMP1, dentin matrix protein 1; MLPA, multiplex ligation dependent probe amplification; NIPT, non-invasive prenatal test; VLK, vertebrate lonesome kinase; DMP4, dentin matrix protein 4; G-CK, Golgi Casein Kinase.
