# Supplementary figures and images for: Compound heterozygous FAM20C gene variants in a patient with severe Raine syndrome: a case report
Source: Front Genet. 2023 Apr 26;14:1179163. doi: 10.3389/fgene.2023.1179163 (PMC10171555; doi:10.3389/fgene.2023.1179163)

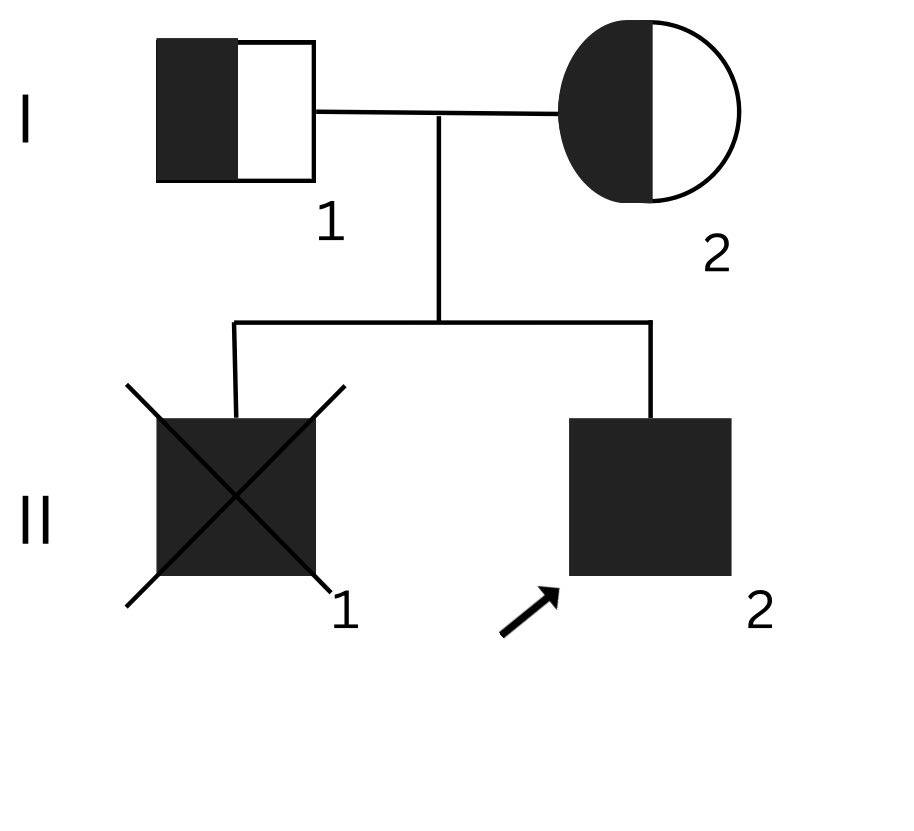

Supplement: Supplementary file 1 [file Image1.JPEG]
